# Structural Features of a Bacteroidetes-Affiliated Cellulase Linked with a Polysaccharide Utilization Locus

**DOI:** 10.1038/srep11666

**Published:** 2015-07-02

**Authors:** A.E. Naas, A.K. MacKenzie, B. Dalhus, V.G.H. Eijsink, P.B. Pope

**Affiliations:** 1 Department of Chemistry, Biotechnology and Food Science, Norwegian University of Life Sciences, Ås, 1432 NORWAY; 2 Department of Medical Biochemistry, Institute for Clinical Medicine, University Of Oslo, PO Box 4950, Nydalen, N-0424, Oslo, NORWAY; 3 Department of Microbiology, Clinic for Diagnostics and Intervention, Oslo University Hospital, Rikshospitalet, PO Box 4950, Nydalen, N-0424, Oslo, NORWAY

## Abstract

Previous gene-centric analysis of a cow rumen metagenome revealed the first potentially cellulolytic polysaccharide utilization locus, of which the main catalytic enzyme (*AC2a*Cel5A) was identified as a glycoside hydrolase (GH) family 5 endo-cellulase. Here we present the 1.8 Å three-dimensional structure of *AC2a*Cel5A, and characterization of its enzymatic activities. The enzyme possesses the archetypical (β/α)_8_-barrel found throughout the GH5 family, and contains the two strictly conserved catalytic glutamates located at the C-terminal ends of β-strands 4 and 7. The enzyme is active on insoluble cellulose and acts exclusively on linear β-(1,4)-linked glucans. Co-crystallization of a catalytically inactive mutant with substrate yielded a 2.4 Å structure showing cellotriose bound in the −3 to −1 subsites. Additional electron density was observed between Trp178 and Trp254, two residues that form a hydrophobic “clamp”, potentially interacting with sugars at the +1 and +2 subsites. The enzyme’s active-site cleft was narrower compared to the closest structural relatives, which in contrast to *AC2a*Cel5A, are also active on xylans, mannans and/or xyloglucans. Interestingly, the structure and function of this enzyme seem adapted to less-substituted substrates such as cellulose, presumably due to the insufficient space to accommodate the side-chains of branched glucans in the active-site cleft.

Cellulose from lignocellulosic biomass holds great potential as a future energy source, given its energy-rich composition and abundance on earth. However, accessing the β-(1,4)-linked glucose units of the cellulose polymer is complicated by the recalcitrant nature of its crystalline molecular structure, which still poses a major obstacle in the efficient industrial production of biofuels from plant biomass^1^,^2^. Overcoming these issues relies on obtaining a complete understanding of how this conversion efficiently occurs in nature, as well as increasing our knowledge and arsenal of cellulolytic enzymes and microbial cellulolytic systems.

The current perception of microbial cellulose-degradation involves two major accepted paradigms, namely secreted endo- and exocellulase enzymes in aerobic micro-organisms, or dockerin-bound endo- and exocellulases arranged in cellulosomes in anaerobes^3^. Recently, other scenarios have been identified. These include the discovery of free enzymes in anaerobic, cellulosome-containing systems^4^,^5^ as well as an apparently intermediate strategy employed by the thermophillic bacterium *Caldicellulosiruptor bescii,* which produces a highly effective cellulase composed of several catalytic domains with complementary functions^6^. Another potentially novel mechanism concerns the presence of a cellulose-targeting Polysaccharide Utilization Locus (PUL) identified in an uncultured Bacteroidetes phylotype (*AC2a*) from the cow rumen^7^. A PUL-based mechanism^8^,^9^,^10^ relies on cellulose binding proteins and cellulases which are tethered to the outer membrane of the bacterium. Since the *AC2a* PUL is the first cellulose-targeting PUL described^7^, further characterization of its cellulases and their activity on crystalline cellulose is of interest. We have studied one of these cellulases, a member of the glycoside hydrolase family 5 (GH5), referred to as *AC2a*Cel5A.

Enzymes classified as GH5 in the Carbohydrate Active Enzymes database (CAZy) are functionally diverse with 20 experimentally verified activities on various polysaccharides, including xyloglucans, mannans, mixed-linkage β-glucans and cellulose (β-1,4 linked glucose)^11^. GH5s can be further divided into subfamilies based on their sequence similarity and enzyme specificities^12^. *AC2a*Cel5A is affiliated with subfamily 4, a polyspecific family which typically includes extracellular bacterial enzymes that exhibit one or more activities categorized as endoglucanase, xyloglucan-specific endoglucanase, xylanase, and licheninase. GH5 enzymes share a common (β/α)_8_-barrel fold, which contains two conserved catalytic glutamic acid residues at the C-terminal ends of the fourth and seventh β-strands. As endo-cellulases are important constituents in commercial cellulase mixtures, the discovery and characterization of additional cellulolytic GH5 enzymes from previously non-accessible biodiversity may prove fruitful for the development of better biomass conversion technology.

Here, we present the three dimensional structure and biochemical characterization of *AC2a*Cel5A. The PUL-derived GH5 cellulase was discovered in the genome of an uncultured Bacteroidetes phylotype reconstructed from a cow rumen metagenome, which was previously assigned to the order Bacteroidales by genome-wide alignment against the NCBI^13^,^14^. The gene was synthesized for expression in *Escherichia coli* with a C-terminal His_6_*-*tag for ease of purification. The native structure was solved to 1.8 Å resolution and revealed a narrow substrate-cleft, likely determining the specificity of the enzyme for linear and less substituted substrates.

## Results

### Structural features of *AC2a*Cel5A

**Figure 1:**
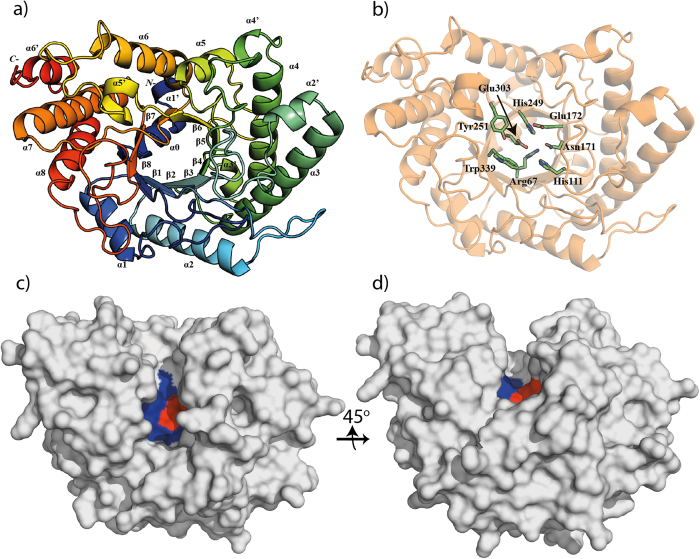
Overall structure and features of *AC2a*Cel5A. **a**) Cartoon representation, rainbow colored blue to red from residue 9 to residue 397. Canonical secondary structure elements are labeled as **α1–α8** for alpha-helices, and **β1–β8** for beta-sheets. The conserved additional N- terminal helix is labeled **α0,** and the six remaining helices additional to the canonical fold are labeled **α1′–α6′**. **b**) Overall structure highlighting the side-chains of strictly conserved residues in the catalytic centers of family 5 GHs, including the catalytic acid, Glu172 and the catalytic nucleophile/base, Glu303^15^,^41^. **c,d)** Surface-views highlighting the active-site groove. The catalytic glutamates are colored red, and other strictly conserved residues highlighted in panel b are colored blue.

Wild-type *AC2a*Cel5A contains a predicted N-terminal signal peptide, which was removed to simplify over-expression of the 405-residue recombinant protein, including mature *AC2a*Cel5A (397 residues) followed by an eight residue C-terminal His_6_-tag. The structure of *AC2a*Cel5A was solved to 1.8 Å, and contained two molecules in the asymmetric unit. The final model comprised residues 9–397; no electron density was observed for the nine N-terminal residues nor for the final eight C-terminal residues, including the His_6_-tag. The data collection and refinement parameters are summarized in Table 1. The enzyme displays the typical (β/α)_8_ barrel structure associated with the GH5 family (Fig. 1a). This canonical (β/α)_8_ fold is preceded by an extra alpha-helix (Fig. 1a), as was also observed in its close structural homologues, cellulase EngD from *Clostridium cellulovorans* (PDB id 3NDY)^15^ and CelAcd from *Piromyces rhizinflata* (PDB id 3AYR)^16^. Additionally, the structure includes six short helices, one in the N-terminus and five in the loop regions between the canonical strands and helices (Fig. 1a; α1′–α6′).

The six closest structural homologues, which include all the biochemically characterized members of subfamily GH5_4, were identified using the Dali server^17^ and were used to produce a structure-based multiple sequence alignment using the PROMALS3D server^18^ (Supplementary Fig. S1). This allowed identification of the conserved catalytic residues, i.e. Glu172 and Glu303, acting as the catalytic acid and nucleophile/base, respectively. Six other strictly conserved residues in the catalytic centers of GH5s^15^ were also identified (Fig. 1b). A surface projection shows the narrow groove stretching across the enzyme, with the active-site located in its center (Fig. 1c–d). The roles of Glu172 and Glu303 as catalytic residues were confirmed by site-directed mutagenesis experiments, showing that *AC2a*Cel5A_E_E172A_ and *AC2a*Cel5A_E_E303A_ were both inactive in the standard carboxymethylcellulose (CMC) assay.

### Cellotriose complex

**Figure 2:**
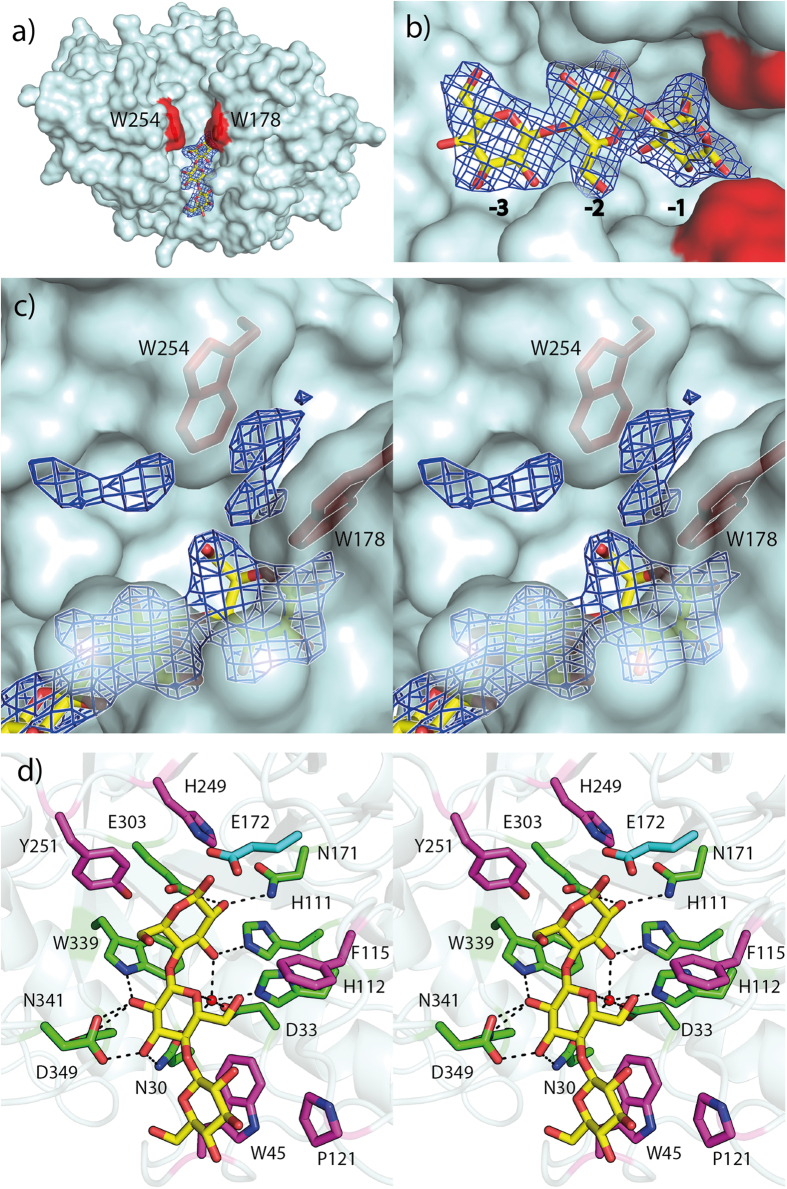
Structure of *AC2a*Cel5A in complex with cellotriose. **a**) Surface view showing cellotriose (yellow carbons) in the active site-cleft with its 2Fo − Fc map contoured at 1.0 σ. The aromatic residues Trp178 and Trp254 are indicated in red to demonstrate their positions in the cleft. **b**) Close-up view of the bound cellotriose with its 2Fo − Fc map contoured at 1.0 σ, indicating the −3 to −1 subsites **c**) Divergent (wall-eyed) stereo view detailing un-modelled 2Fo − Fc electron density in the cleft contoured at 1.0 σ. The side chains of Trp178 and Trp254 are shown as sticks and colored red. **d**) Divergent (wall-eyed) stereo view of ligand-protein interactions determined using LigPlot+. Green residues hydrogen-bond to cellotriose (yellow), whereas magenta residues interact with the ligand by hydrophobic and Van der Waals forces. The position of the side chain of Glu172 (cyan) was determined by superposition of the wild-type structure.

Co-crystallization of the E172A mutant with cellotetraose resulted in the complex structure solved to 2.4 Å resolution, with two molecules in the asymmetric unit. Additional electron density was observed in the active-site cleft of molecule A, and was refined as a cellotriose molecule bound to the −3 to −1 subsites (Fig. 2a–b). No electron-density for a bound ligand could be observed in the B molecule, which also lacked electron density for several main-chain residues, namely 34–45 and 112–127. These residues correspond to the loop regions between β1 and α1, and between β3 and α2’, respectively, and have been omitted from the final model. Even though the ligand density appears to fit a cellotriose molecule, it is likely that a cellotetraose molecule is present in the complex as the enzyme only produces dimers from cellotetraose^7^. The observed cellotriose density could reflect three glucose moieties of a tetramer, where the fourth moiety could be positioned in the putative −4 position and be disordered. This is supported by the observation that the electron density of the -3 bound moiety is incomplete towards O4 (Fig. 2b).

Furthermore, in both the apo- and the complex structure, extra electron density was also observed close to, and between Trp178 and Trp254 in the substrate-binding cleft of both chain A and B (Fig. 2c). The electron density was insufficient to allow meaningful interpretation, but did not appear to resemble any of the components found in the cryoprotectant or mother liquor. In GH5 complexes where either a tetramer or pentamer ligand is bound across the catalytic site, a tryptophan corresponding to Trp178 of *AC2a*Cel5A is always interacting with the sugar moiety bound in the +1 subsite and in some cases also the sugar in the +2 subsite (PDB ids; 4HU0, 3AZT^19^, 2CKR, 1H5V, 1ECE^20^, 3QHN^21^ and 4OOZ^22^). The aromatic residue corresponding to Trp254 in *AC2a*Cel5A is much less conserved; this residue’s role is most commonly appears to be taken by tyrosine (2CKR, 1ECE, 3QHN), whereas also proline (4HU0), phenylalanine (3AZT) and histidine (4OOZ) occur in a similar position of the active-site cleft. 1H5V lacks a residue corresponding to Trp254. While the role of the Trp178-Trp254 “clamp” in ligand binding seems obvious, it seems unlikely that the density, observed in both the apo- and the ligand structure, reflects substrate. It is worth noting that the C-terminus of an adjacent B molecule is located in close proximity to the active-site cleft of molecule A, and vice versa. It is therefore likely that the electron density in the Trp-Trp “clamp”, as well as the additional density seen outside the clamp, reflects a non-specific interaction of the C-terminal His_6_–tag which protrudes towards the active site cleft.

LigPlot+^23^ was used to investigate the interactions of the enzyme with the cellotriose molecule, and the estimated interactions are shown in Fig. 2d. The glucose moiety bound in the −1 subsite is stabilized by hydrogen bonding from its O2 group to the catalytic Glu303 residue, as well as the strictly conserved Asn171. O3 interacts with His111 as well as Asn30, Asp33 and His112 via a water molecule, HOH25. The same water also interacts with O5 of the glucose bound in the -2 subsite. The O2 of the latter sugar has hydrogen-bond type interactions with Trp339, Asn341 and Asp349; Asp349 also interacts with the O3 together with Asn30. Unlike the sugars bound in the −2 and −1 subsites, the sugar bound in the −3 subsite involves no hydrogen bonds between the ligand and the protein. Its binding seems to be mediated by stacking interactions with Trp45. Other hydrophobic and van der Waals contacts contributing to ligand binding involve the strictly conserved His249 and Tyr251, which interact with the −1 glucose moiety, and Phe115, interacting with the −2 sugar. Superposition of the native structure on the cellotriose complex placed a carboxyl oxygen of the catalytic acid, Glu172, at 1.7 Å of O1 of the glucose bound in the −1 subsite.

### Biochemical characterization

**Figure 3:**
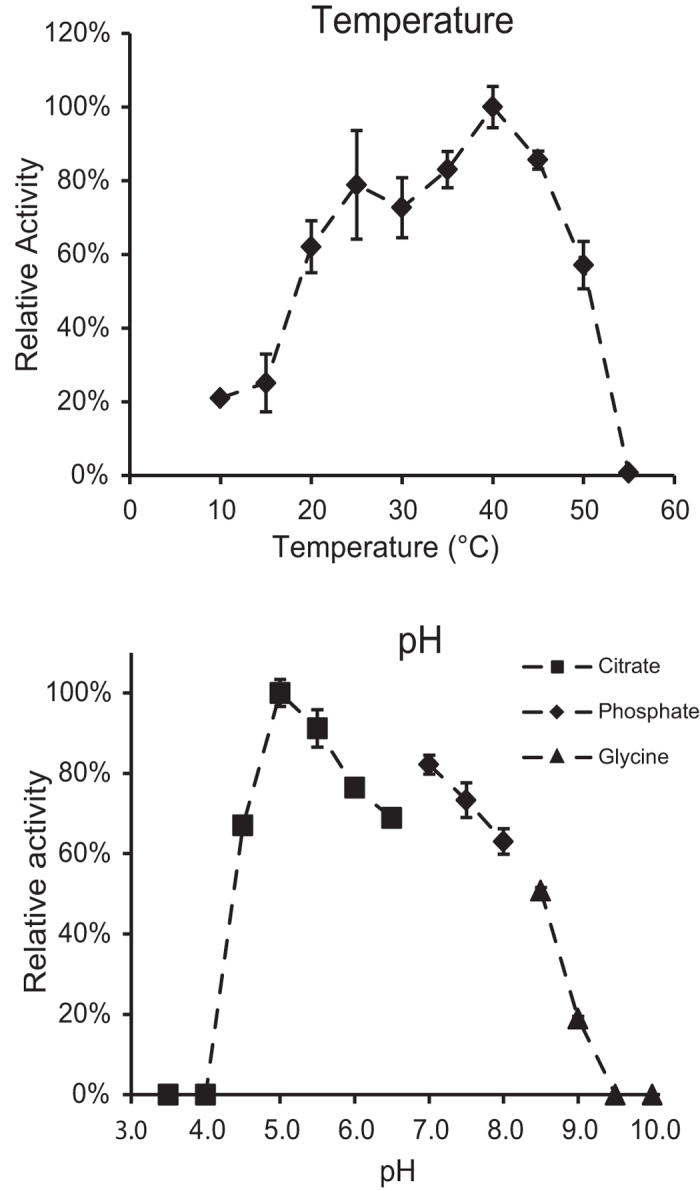
Biochemical characterization of *AC2a*Cel5A. Enzyme activity is reported as the amount of reducing sugars released in the standard CMC assay, relative to optimal temperature (top panel) or pH (bottom panel) conditions. Error bars represent standard deviations between three replicates.

As previously reported^7^, *AC2a*Cel5A is an endoglucanase active on *β*-(1,4) glycosidic bonds between glucose units in soluble and insoluble cellulosic substrates and in linear β-glucans. Further characterization and quantification of enzyme activities revealed high activities on linear β-(1,4) glucans, whereas the enzyme displayed only trace activity on soluble tamarind xyloglucan and no activity could be detected on solubilized Birchwood xylan (Table 2). Using the standard CMC assay, the optimum temperature of the enzyme was found to be 40 °C, correlating with the 38–40 °C environment of the cow rumen^24^ (Fig. 3). However, the enzyme showed activity over a relatively broad temperature range, retaining close to 60% of maximum activity between 20 °C and 50 °C. Assays with different buffer systems of various pH values also showed a broad activity range. Maximum activity was observed at pH 5.0, while over 60% of maximum activity was maintained between pH 4.5 and 8.0.

### Comparison to structural homologues

The six closest structural homologues of *AC2a*Cel5A previously identified with the Dali server were subjected to further investigation (Table 2). The selected structures had root-mean-squared deviations (R.m.s.d.) of less than 1.9 Å when compared with *AC2a*Cel5A. Two of the homologues have been characterized as xyloglucanases (PDB ids: 2JEP and 3ZMR), whereas the four others are identified as cellulases (PDB ids: 3NDY, 4IM4, 1EDG and 3AYR) (Table 2). The xyloglucanase homologues are reported as strictly specific for xyloglucan, whereas the cellulase-type homologues generally seem more promiscuous in their substrate specificities. For example, EngD (PDB id 3NDY) has comparable activity on xyloglucan (1,6-xylose substituted β-1,4 glucan), CMC and Barley β-glucan, and also showed activity on birchwood xylan (β -1,4 xylose substituted with 10.2% hexuronic acids^25^). CelCCa (PDB id 1EDG) from *Clostridium thermocellum* and CelAcd (PDB id 3AYR) from *Piromyces rhizinflata* were not tested on xyloglucan, but showed activities on insoluble xylan from Sigma, and soluble oat spelt xylan (substituted with arabinose (9%), glucose (7%), galactose (1%)^25^), respectively. Conversely, *AC2a*Cel5A is specific for unsubstituted β-1,4 glucans and CMC (cellulose substituted with relatively small carboxymethyl groups), with only trace activity on xyloglucan and no detectable activity on birchwood xylan.

**Figure 4:**
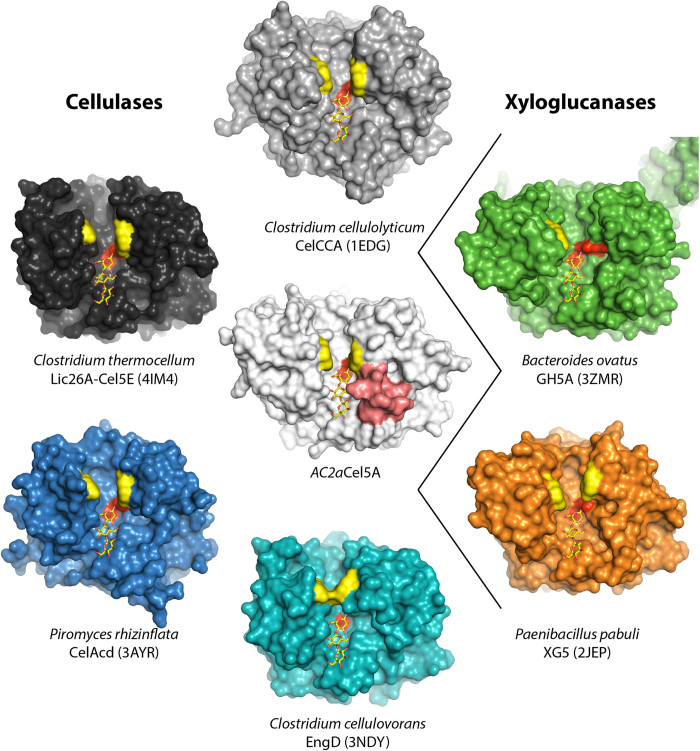
Active site grooves in related GH5 endoglucanases. Catalytic centers are indicated by the two catalytic glutamates whose surfaces are colored red. The surface of the residues lining the +1 and/or +2 subsites, analogous to Trp178 and Trp254 in *AC2a*Cel5A are colored yellow (see text for details). The 115-126 loop in *AC2a*Cel5A is coloured red. The −3 to −1 subsites are indicated by the superimposed cellotriose molecule from *AC2a*Cel5A.

**Figure 5:**
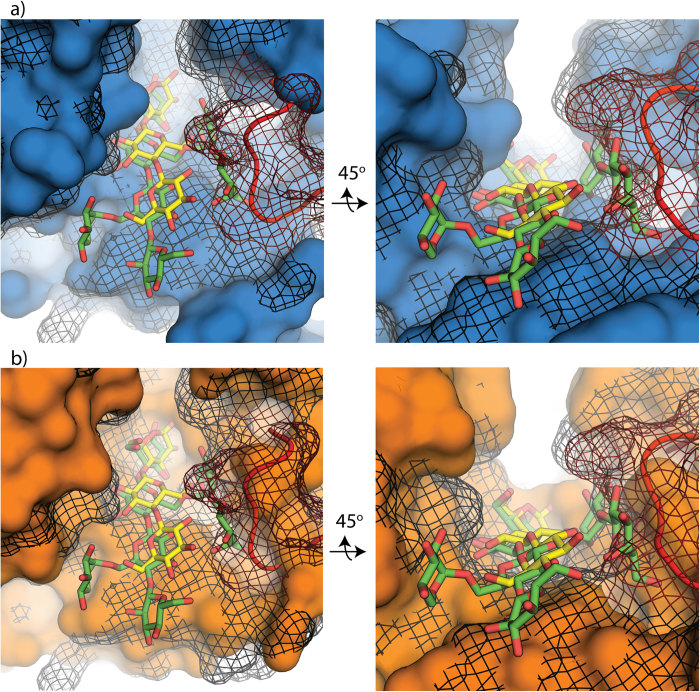
Structural superpositions. **a**) CelAcd from *P. rhizinflata* (PDB id 3AYS) (blue) structurally aligned by PyMOL to *AC2a*Cel5A (black mesh). The main chain of the loop region of *AC2a*Cel5A between β3 and α2’ is shown and colored red. The ligands shown are cellotriose (yellow) binding to the −3 to −1 site of *AC2a*Cel5A and a xyloglucan tetramer (green) which was positioned by structural superposition of the ligand-containing structure of *P. pabuli* XG5 xyloglucanase (PDB id 2JEQ). The xyloglucan ligand is an XXLG tetramer, where G, X and L denote unsubstituted _D_-Glc*p,* α-_D_-Xyl*p*-(1,6)-_D_-Glc*p*, and β-_D_-Gal*p*-(1,2)-α-_D_-Xyl*p*(1,6)-_D_-Glc*p*, respectively. **b**) Shows the same structural alignment as in panel a), with the same elements and coloring, but with the *P. pabuli* XG5 xyloglucanase, shown in orange, instead of CelAcd. The xyloglucan tetramer binds to subsites −1 to −4.

A comparison of the active-site clefts of the GH5s shows that *AC2a*Cel5A has a narrower active site cleft than its homologues, especially in the vicinity of the −3 to −1 sub-sites (Fig. 4). This is primarily caused by the loop region between β3 and α3’ (red surface), which protrudes into the substrate-binding cleft and which is extended in *AC2a*Cel5A, compared to the examined homologues (Supplementary Fig. S1, residues 115–126). The figure also indicates the aromatic residues, corresponding to the putative +1 site of *AC2a*Cel5A, that are structurally conserved in these close homologues (yellow surface). Fig. 5a shows a detailed view of the differences between the surface projections of *AC2a*Cel5A (black mesh) and the cellulase CelAcd (blue), highlighting that this homologue has a wider active site cleft. The picture further shows that the active site cleft of *AC2a*Cel5A is far too narrow to accommodate a xyloglucan tetramer due to the extended loop between β3 and α3’ (red backbone and mesh), whereas CelAcd has enough space to accommodate this branched oligosaccharide. Fig. 5b shows that a true xyloglucanase such as XG5 from *Paenibacillus pabuli* also has a wider cleft than *AC2a*Cel5A but that this cleft is narrower compared to the promiscuous CelAcd. In the XG5 xyloglucanase, the cleft seems more optimally shaped to harbour the xyloglucan fragment, providing many tight interactions.

## Discussion

*AC2a*Cel5A is an endo-acting cellulase that is encoded within the first reported cellulolytic PUL, originating from an uncultured Bacteroidetes phylotype. Biochemical data presented in this study demonstrate that optimal conditions for *AC2a*Cel5A activity correspond well with the cow rumen environment. *AC2a*Cel5A was evolutionarily distinct compared to its nearest structural homologues (30–34% sequence identity, Table 2), whereas its nearest sequence homologue was a putative GH5 recovered from a goat rumen metagenome (51% sequence identity, accession number: AIF26005). According to the CAZy subfamily classification proposed by Aspeborg *et al.*^12^, *AC2a*Cel5A and all of the examined homologues (Table 2) belong to subfamily GH5_4. Among these subfamily GH5_4 enzymes, the deeply branched *AC2a*Cel5A is dissimilar in that it lacks activity on both xylan and xyloglucan. The interactions between cellotriose and the -3 to -1 subsites of *AC2a*Cel5A are similar to the interactions observed in enzyme-substrate complexes of EngD^15^ (PDB id 3NDZ) and CelAcd^16^ (PDB id 3AYS). Nonetheless, *AC2a*Cel5A is unique in that it has an exceptionally narrow active site cleft, due to an extended loop region that seems well adapted to (only) acting on less-substituted β-glucans.

*AC2a*Cel5A contains a tryptophan “clamp” in its putative +1 and +2 subsites, analogous to what has been observed in other glycoside hydrolases acting on insoluble polysaccharides^26^,^27^. This aromatic pair is not conserved in all GH5s (see text above), but all of the examined structural homologues except the *Bacteroides ovatus* xyloglucanase, *Bo*GH5A (PDB id 3ZMR), do contain two structurally conserved aromatic residues that form a similar substrate-binding “clamp” (Fig. 4). Although the complex structure of *AC2a*Cel5A does not provide direct new insight into the interaction between the two aromatic residues and the substrate, the presence of electron density in the “clamp” in both the apo and complex structures suggests a potential for strong stacking interactions, and likely indicates the enzyme’s putative +1 (and +2) subsites.

This study presents new insights into the biology of an as-yet uncultured Bacteroidetes-affiliated phylotype (*AC2a*). Collectively, the *AC2a*Cel5A enzyme structure and activity data suggest specificity towards linear and less substituted β-(1,4)-linked glucans, and strengthens previous hypotheses that the PUL encoding *AC2a*Cel5A is cellulolytic. Ongoing studies directed towards elucidating the structures and functions of other proteins encoded by this PUL (e.g. SusD and SusE cellulose-binding proteins) will enhance our understanding of its polysaccharide degradation potential.

## Methods

### Protein discovery and expression

The GH5 enzyme (*AC2a*Cel5A) was identified and produced as previously described^7^. In brief, the gene was synthesized without its predicted signal peptide (residues 1–18), cloned into the pNIC-CH vector^28^ and over-expressed in *Escherichia coli*. The C-terminally His_6_-tagged protein was purified to near homogeneity by immobilized metal affinity chromatography, and the purity was assessed by SDS-PAGE. The protein concentration was estimated by A_280_ using the molar extinction coefficient calculated from the protein sequence. The protein was stable at concentrations around 20 mg/ml in 20 mM Tris-HCl pH 8 with 0.2 M NaCl, at 4 °C, for several months.

### Site-directed mutagenesis

The QuickChange II site-directed mutagenesis kit (Agilent) was used to mutate the catalytic glutamic acid residues (E172 and E303) to alanines, using the following primers; E172AF; GGT TTT TGA AAC CCT GAA TGC AAT TCA GGA TGG TGA TTG GG, E172AR; CCC AAT CAC CAT CCT GAA TTG CAT TCA GGG TTT CAA AAA CC, E303AF; GCC GGT TTA TGG TGC ATT TGG TGC CGT TCG, E303AR; ACG AAC GGC ACC AAA TGC ACC AAA ATA AAC CGG.

### Crystallization, diffraction data collection and structure determination

Native crystals were produced by screening sitting drop vapor diffusion conditions using the JCSG+ 1 kit (Molecular Dimensions; Altamonte Springs, FL, USA), with a reservoir volume of 100 μL, and a drop comprising 0.5 μL well solution +0.5 μl purified protein solution (10.2 mg/ml in 20 mM Tris-HCl pH 8.0, 0.2 M NaCl). Crystals grew overnight in several conditions, with highest quality crystals forming in conditions consisting of 0.1 M sodium cacodylate pH 6.5, 40% v/v 2-Methyl-2,4-pentanediol and 5% w/v PEG8000. Crystals for the ligand bound complex were obtained by co-crystallization of the catalytically inactive *AC2a*Cel5A E172A mutant in conditions consisting of 0.2 M potassium nitrate and 20% w/v PEG 3350. The reservoir volume was 200 μL and the 2 μL drop contained a 1:1 mixture of well solution and protein solution (11 mg/ml protein in 20  mM Tris-HCl pH 8.0, 0.2 M NaCl, 5 mM cellotetraose). Crystals were mounted in loops and flash-frozen with liquid nitrogen. X-ray diffraction data was collected at the ID29 beamline at the European Synchrotron Radiation Facility (ESRF), to a resolution of 1.8 Å for the apo protein, and 2.1 Å for the complex. The apo data was processed using iMOSFLM^29^, Aimless^30^ and tools in the CCP4i package^31^. The structure was solved by molecular replacement with Phaser^32^ using a poly-alanine model of the structure of a GH5 family xyloglucanase from *Paenibacillus pabuli*^33^ (PDB-entry 2JEP). The initial model was built using the autobuild function of PHENIX^34^ and further refined using PHENIX, RefMac5^35^,^36^ and manual rebuilding in Coot^37^. The dataset for the complex was processed with the XDS package^38^ and scaled using Scala^39^, cutting the data to 2.4 Å. The structure was solved by molecular replacement using the apo structure of *AC2a*Cel5A with MolRep, and the structure was refined using RefMac5 and manual rebuilding in Coot. All protein structure figures were produced in PyMOL.

### Enzyme characterization

The substrate specificity of *AC2a*Cel5A had previously been determined using Azurine-Crosslinked Polysaccharide (AZCL) substrates, along with activity on insoluble cellulose substrates^7^. In this study, further characterization of the enzymatic activity was performed using the soluble β-(1,4) linked glucan substrates carboxymethylcelullose (CMC) (Sigma-Aldrich), barley β-glucan (Megazyme), tamarind xyloglucan (Megazyme), lichenan (Sigma-Aldrich), and Birchwood Xylan (Carl Roth). The standard reaction using CMC contained 20 mM BisTris buffer pH 6.5, 25 nM enzyme, 20 mM CaCl_2_, and 10 mg/ml substrate in a total volume of 200 μl. Enzyme was added to pre-heated assay mixtures and the reactions were incubated at 40 °C, with 900 rpm vertical shaking. 100 μl sample was taken after 10 minutes, and added to 100 μL DNS reagent^40^. The amount of reducing ends released were determined as glucose equivalents using the DNS reducing-end assay and a glucose standard curve. Assays for activity on barley β-glucan, lichenan, xylan and xyloglucan were performed with 0.5% substrate (w/v), and 10, 10, 200 and 200 nM enzyme load, respectively. A Unit of enzyme activity was defined as the amount of enzyme releasing one μmol of glucose equivalents per minute.

## Additional Information

**How to cite this article**: Naas, A.E. *et al.* Structural Features of a Bacteroidetes-Affiliated Cellulase Linked with a Polysaccharide Utilization Locus. *Sci. Rep.* **5**, 11666; doi: 10.1038/srep11666 (2015).

## Supplementary Material

Supplementary Information

## Figures and Tables

**Table 1:** Crystal data, data-collection statistics and refinement data.

Dataset	*AC2a*GH5 native structure	*AC2a*GH5_E172A cellotriose complex
PDB Code	4YHE	4YHG
Data Collection		
Beamline	ID29 (ESRF)	ID29 (ESRF)
Wavelength (λ)	0.9724	0.9791
Space Group	P 2_1_ 2_1_ 2_1_	P 2_1_ 2_1_ 2_1_
Unit-cell parameters (Å, °)	a = 85.5, b = 100.7, c = 101.9	a = 84.3, b = 99.6, c = 102.5
	α = β = γ = 90	α = β = γ = 90
Resolution (Å)	65.5 - 1.85 (1.9 - 1.85)	54.5-7.6 (2.5-2.40)
Unique reflections	74 499 (4027)	34 325 (4927)
Multiplicity	3.7 (2.9)	5.2 (5.1)
Completeness (%)	98.6 (90.8)	99.8 (99.8)
Mean I/σI	8.9 (2.9)	10.2 (3.1)
R_meas_^a^	0.094 (0.36)	0.116 (0.551)
Refinement statistics		
R_cryst_/R_free_ (%)^b^	17.8 (21.2)	18.5 (22.8)
R.m.s.d. bonds (Å)	0.011	0.010
R.m.s.d. angles (°)	1.4	1.3
B-factor		
protein/solvent/ligands (Å^2^)	25.0 / 35.5	42.3 / 35.0 / 55.2
Number of atoms		
Protein	6155	5941
Solvent	955	232
Cellotriose		34
Ramachandran plot (%)^c^		
Favorable regions	97.8	97.2
Additionally allowed regions	1.9	2.5
Outliers	0.3	0.3

Values in parentheses are for the highest resolution shells

^a^Rmeas defined by Diederichs & Karplus^42^.

^b^Rcryst  =  Σhkl ||*F*_o_| - |*F*_c_|| / Σ_hkl_|| *F*o | where *F*o and *F*c are the observed and calculated structure factor amplitudes, respectively. *R*_free_ is calculated from a randomly chosen 5% set of all unique reflections not used in refinement.

^c^Defined using MolProbity^43^.

**Table 2:** Structural comparison of *AC2a*Cel5A with its six closest structural homologues identified using the DALI server^17^, along with reported enzyme activities.

Structural comparison				Enzymatic activities							
	Dali Z-score	RMSD (Å)	% Id	CMC (U/mg)	β-glucan (U/mg)	Filter paper (U/mg)	Avicel (U/mg)	Lichenan (U/mg)	Xylan (U/mg)	Xyloglucan (U/mg)	Reference
*Bacteroidetes AC2a* Cellulase Cel5A	−	−	−	216.8	1471.4	0.152^a^	0.115^a^	839.9	nd	trace	*This study* and 7
*Paenibacillus pabuli* Xyloglucanase XG5 (2JEP)	49.2	1.6	34	nd	nd	−	nd	nd	nd	8700	33
*Clostridium cellulovorans C*ellulase EngD (3NDY)	47.0	1.7	33	15	42	−	0.017	−	0.5	36	15
*Bacteroides ovatus* Xyloglucanase BoGH5A (3ZMR)	46.6	1.9	32	nd	nd	−	−	nd	−	514.2	44
*Clostridium thermocellum*Cellulase Lic26A-5E (4IM4)	46.0	1.6	32	Active	1200	−	−	−	Active	−	To be published, NZYTech^b^
*Clostridium cellulolyticum* Cellulase CelCCA (1EDG)	45.9	1.9	32	101.3	104.3	−	0.028 (0.124)^d^	79.2	10.1	−	45,c
*Piromyces rhizinflata* Cellulase CelAcd (3AYR)	43.2	1.8	30	344.9	576	0.64	1.39	542.5	106.2	−	47

RMSD; root-mean-square deviation of C-alpha atoms. The structural homologues are sorted based on the Z-score obtained in the DALI search. One Unit of enzyme activity was defined as the amount of enzyme releasing 1 μmol of reducing sugar equivalents per minute. “nd” means not detected, whereas a hyphen, “-”, indicates “not tested”.

^a^μmol reducing sugar equivalents calculated as μmol cellotriose + μmol cellobiose + μmol glucose, quantified by HPAEC-PAD^7^.

^b^GH5 domain from *Ct*Lic26A-Cel5E. Described as multi-functional cellulase, mannanase and xylanase by Bianchetti *et al.* in the PDB entry file; β-glucan activity for the Cel5E domain reported by NZYTech (1649-038 Lisboa, Portugal/www.nzytech.com).

^c^U/mg calculated from U/μmol reported in^45^.

^d^For this enzyme data for both the full length protein (in parentheses), and the catalytic domain only were published.
