# Author Correction: Structural Features of a Bacteroidetes-Affiliated Cellulase Linked with a Polysaccharide Utilization Locus

**DOI:** 10.1038/s41598-020-62786-2

**Published:** 2020-04-08

**Authors:** A. E. Naas, A. K. MacKenzie, B. Dalhus, V. G. H. Eijsink, P. B. Pope

**Affiliations:** 1 Department of Chemistry, Biotechnology and Food Science, Norwegian University of Life Sciences, Ås, 1432 Norway; 2 Department of Medical Biochemistry, Institute for Clinical Medicine, University Of Oslo, PO Box 4950, Nydalen, N-0424 Oslo Norway; 3 Department of Microbiology, Clinic for Diagnostics and Intervention, Oslo University Hospital, Rikshospitalet, PO Box 4950, Nydalen, N-0424 Oslo Norway

Correction to: *Scientific Reports* 10.1038/srep11666, published online 02 July 2015

This Article contains an error, where the protein name corresponding to 4IM4 (pdbid) is incorrectly given as “Lic26A-Cel5E”, and should read “CelE”.

In addition, three references were omitted from Table 2, and are given below as References 1–3.

The correct Table 2 and Figure 4 are given below as Table 1 and Figure 1 respectively.

**Table 1:** Structural comparison of *AC2a*Cel5A with its six closest structural homologues identified using the DALI server^17^, along with reported enzyme activities.

	Structural comparison			Enzymatic activities							Reference
	DALI Z-score	RMSD (Å)	% id	CMC (U/mg)	β-glucan (U/mg)	Filter paper (U/mg)	Avicel (U/mg)	Lichenan (U/mg)	Xylan (U/mg)	Xyloglucan (U/mg)	
*Bacteroidetes AC2a* Cellulase Cel5A	—	—	—	216.8	1471.4	0.152^a^	0.115^a^	839.9	nd	trace	*This study* and^7^
*Paenibacillus pabuli* Xyloglucanase XG5 (2JEP)	49.2	1.6	34	nd	nd	—	nd	nd	nd	8700	^33^
*Clostridium cellulovorans C*ellulase EngD (3NDY)	47.0	1.7	33	15	42	—	0.017	—	0.5	36	^15^
*Bacteroides ovatus* Xyloglucanase BoGH5A (3ZMR)	46.6	1.9	32	nd	nd	—	—	nd	—	514.2	^44^
*Clostridium thermocellum* Cellulase CelE (4IM4)	46.0	1.6	32	Active	75.4	Active	—	—	Active	—	^1–3^, b
*Clostridium cellulolyticum* Cellulase CelCCA (1EDG)	45.9	1.9	32	101.3	104.3	—	0.028 (0.124)^d^	79.2	10.1	—	^45,46^, c
*Piromyces rhizinflata* Cellulase CelAcd (3AYR)	43.2	1.8	30	344.9	576	0.64	1.39	542.5	106.2	—	^47^

RMSD; root-mean-square deviation of C-alpha atoms. The structural homologues are sorted based on the Z-score obtained in the DALI search. One Unit of enzyme activity was defined as the amount of enzyme releasing 1 µmol of reducing sugar equivalents per minute. “nd” means not detected, whereas a hyphen, “—”, indicates “not tested”.

^a^µmol reducing sugar equivalents calculated as µmol cellotriose + µmol cellobiose + µmol glucose, quantified by HPAEC-PAD^7^.

^b^GH5 domain of CelE.

^c^U/mg calculated from U/µmol reported in^45^.

^d^For this enzyme data for both the full length protein (in parentheses), and the catalytic domain only were published.

**Figure 1:**
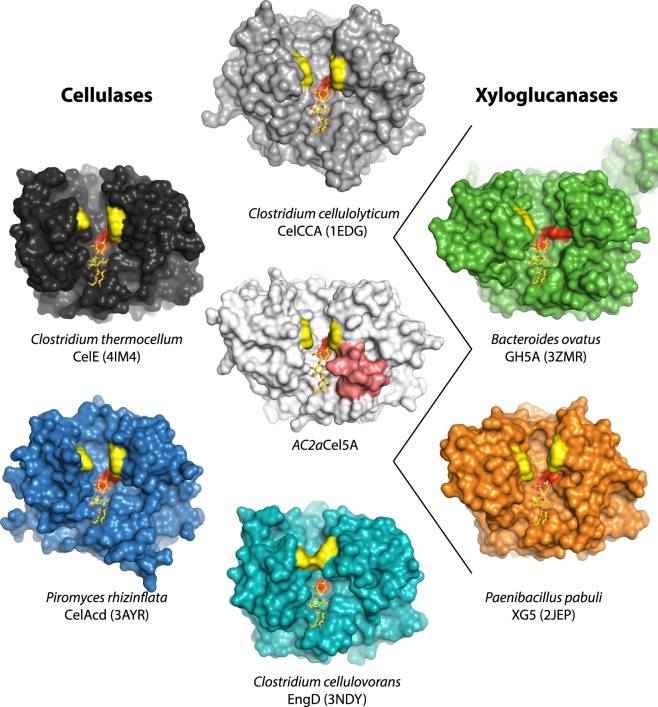
.

Finally, the updated Supplementary Information file, containing the correct protein name in Figure S1, is given below.

## Supplementary information

Supplementary Information.
