# Supplementary material for: Structural Features of a Bacteroidetes-Affiliated Cellulase Linked with a Polysaccharide Utilization Locus
Source: Sci Rep. 2015 Jul 2;5:11666. doi: 10.1038/srep11666 (PMC4488959; doi:10.1038/srep11666)
Supplement: Supplementary Information [file srep11666-s1.pdf]

STRUCTURAL FEATURES OF A BACTEROIDETES-AFFILIATED CELLULASE LINKED WITH A POLYSACCHARIDE UTILIZATION LOCUS

A.E. Naas[1†], A.K. MacKenzie[1], B. Dalhus[2,3], V.G.H. Eijsink[1], P.B. Pope[1†]

1. Department of Chemistry, Biotechnology and Food Science, Norwegian University of Life Sciences, Ås, 1432 NORWAY.
2. Department of Medical Biochemistry, Institute for Clinical Medicine, University Of Oslo, PO Box 4950, Nydalen, N-0424, Oslo, NORWAY
3. Department of Microbiology, Clinic for Diagnostics and Intervention, Oslo University Hospital, Rikshospitalet, PO Box 4950, Nydalen, N-0424, Oslo, NORWAY

†Corresponding Authors: Adrian E. Naas and Phillip B. Pope
Department of Chemistry, Biotechnology and Food Science
Norwegian University of Life Sciences
Post Office Box 5003
1432, Ås
Norway
Phone: +47 6496 6489
Email: adrian.naas@nmbu.no, phil.pope@nmbu.no

Supplementary information

```
                                                   α1′   α0      β1
AC2a_GH5        9  --------------------------------RPMDNEAVQFGMSMGIGWNLGNQMDAHYD--------G
XG5     (2JEP)  1  ------------------------------------ADASQIVSEMGAGWNLGNQLEAAVN--------G
EngD    (3NDY)  1  ---------------------------STAFTGVRDVPAQQIVNEMKVGWNLGNTMDAIG----------
BoGH5A  (3ZMR) 71  VSASGAPDIIINVKQSLYSVPAYDEYIAPDNTGMRDLTSMQLSALMKAGVNVGNTFEAVIVGND---GSL
26A-5E  (4IM4)  1  --------------------------------GMRDISAIDLVKEIKIGWNLGNTLDAP-----------
CelCCA  (1EDG)  1  -----------------MYDASLIPNLQIPQKNIPNNDGMNFVKGLRLGWNLGNTFDAFNGTNI---TNE
CelAcd  (3AYR)  1  -------------------------HHVDDDDKIRDISSKELIKEMNFGWNLGNTMDAQCIEYLNYEKDQ

                                α2       β2                      α3           β3
AC2a_GH5       39  CSYETGWGNKAATQQTFNGLAKAGFRSVRIPVTWMGHIGNAPTYAIERGWLDRVDELVHMAHKAGLIVII
XG5     (2JEP) 27  TPNETAWGNPTVTPELIKKVKAAGFKSIRIPVSYLNNIGSAPNYTINAAWLNRIQQVVDYAYNEGLYVII
EngD    (3NDY) 34  --GETNWGNPMTTHAMINKIKEAGFNTLRLPVTWDGHMGAAPEYTIDQTWMKRVEEIANYAFDNDMYVII
BoGH5A  (3ZMR)138  SGDETCWGNPTPNKVLFEGIKAAGFDVVRIPVAYSHQFEDAATYKIKSAWMDKVEAAVKAALDAGLYVII
26A-5E  (4IM4) 28  --TETAWGNPRTTKAMIEKVREMGFNAVRVPVTWDTHIGPAPDYKIDEAWLNRVEEVVNYVLDCGMYAII
CelCCA  (1EDG) 51  LDYETSWSGIKTTKQMIDAIKQKGFNTVRIPVSWHPHVSG-SDYKISDVWMNRVQEVVNYCIDNKMYVIL
CelAcd  (3AYR) 46  TASETCWGNPKTTEDMFKVLIDNQFNVFRIPTTWSGHFGEAPDYKIDEKWLKRVHEVVDYPYKNGAFVIL
                                           ▲Arg67
                                           α2′      α3                  β4    *
AC2a_GH5      109  NIHHDGFGAADTPSKGSHWLDLPAAVASEERNQLIKQELTMIWLQIGKRFANDGEWLVFETLNEIQDG--
XG5     (2JEP) 97  NIHGDGYNS--VQG---GW-LLV----NGGNQTAIKEKYKKVWQQIATKFSNYNDRLIFESMNEVFDG--
EngD    (3NDY)102  NLHHEN-----------EW-LKP----FYANEAQVKAQLTKVWTQIANNFKKYGDHLIFETMNEPRPVGA
BoGH5A  (3ZMR)208  NIHWEG-----------GWLNHP----VDANKEALDERLEAMWKQIALRFRDYDDRLLFAGTNEVNNDDA
26A-5E  (4IM4) 96  NLHHDN-----------TW-IIP----TYANEQRSKEKLVKVWEQIATRFKDYDDHLLFETMNEPREVGS
CelCCA  (1EDG)120  NTHHDV--------DKVKG-YFP----SSQYMASSKKYITSVWAQIAARFANYDEHLIFEGMNEPRLVGH
CelAcd  (3AYR)116  NLHHE------------TW-NHA----FSETLDTAKEILEKIWSQIAEEFKDYDEHLIFEGLNEPRKNDT
                     ▲His111                                            Asn171▲▲Glu172
                      α3′          α4           α4′  β5        α5
AC2a_GH5      177  --DWGNGNNRRDGGAQYRVLNEWNQVCVDAIRAAAGKNETRYIGVPGYVCNPDLTVE--NLVLPEDV---
XG5     (2JEP)155  --NYGNP-----NSAYYTNLNAYNQIFVDTVRQTGGNNNARWLLVPGWNTNIDYTVGNYGFTLPTDNYRS
EngD    (3NDY)156  SLQWTGG-----SYENREVVNRYNLTAVNAIRATGGNNATRYIMVPTLAASAMSTTIN-DLVIPNN----
BoGH5A  (3ZMR)263  --NGAQP-----TEENYRVQNGFNQVFVNTVRATGGRNHYRHLIVQAYNTDVAKAVA--HFTMPLDI---
26A-5E  (4IM4)150  PMEWMGG-----TYENRDVINRFNLAVVNTIRASGGNNDKRFILVPTNAATGLDVALN-DLVIPNN----
CelCCA  (1EDG)177  ANEWWPELTNSDVVDSINCINQLNQDFVNTVRATGGKNASRYLMCPGYVASPDGATND-YFRMPNDI---
CelAcd  (3AYR)169  PVEWTGG-----DQEGWDAVNAMNAVFLKTVRSAGGNNPKRHLMIPPYAAACNENSFN-NFIFPED----

                             β6       α5′                                 α6
AC2a_GH5      240  -----VPNRLMVAVHSYDPWDYAGSA----KYNEWGHTG---KDVVPGVGEEAYVGMLNRLFNMYIRRGV
XG5     (2JEP)218  SAIPSSQKRIMISAHYYSPWDFAGEEN--GNITQWGATSTNPAKKSTWGQEDYLESQFKSMYDKFVTQGY
EngD    (3NDY)216  ------DSKVIVSLHMYSPYFFAMDIN---GTSSWGS----------DYDKSSLDSEFDAVYNKFVKNGR
BoGH5A  (3ZMR)321  -----VQNRIFLECHYYDPYDFTIMPNDENFKSQWGAAF-AGGDVSATGQEGDIEATLSSL-NVFINNNV
26A-5E  (4IM4)210  ------DSRVIVSIHAYSPYFFAMDVN---GTSYWGS----------DYDKASLTSELDAIYNRFVKNGR
CelCCA  (1EDG)243  SGN---NNKIIVSVHAYCPWNFAGLAMADGGTNAWNIND--------SKDQSEVTWFMDNIYNKYTSRGI
CelAcd  (3AYR)229  ------DDKVIASVHAYAPYNFALNNG-EGAVDKFDA-----------AGKRDLEWNINLMKKRFVDQGI
                              His249▲ ▲Tyr251
                    β7                    α7               β8
                       *
AC2a_GH5      298  PVYFGEFGAVRRA------SKADEEFRLYYFRYICKAMRDRRISALYWDNGNSK-AGNDGFGVIDHATGR
XG5     (2JEP 286  PVVIGEFGSIDKTSY----DSSNNVYRAAYAKAVTAKAKKYKMVPVYWDNGHNG---QHGFALFNRSNNT
EngD    (3NDY)267  AVVIGEMGSINKN---------NTAARVTHAEYYAKSAKARGLTPIWWDNGYSVAGKAETFGIFNRSNLT
BoGH5A  (3ZMR)384  PVIIGEYGPTLRDQLTGEALENHLKSRNDYIEYVVKTCVKNKLVPLYWDAGY-------TEKLFDRTTGQ
26A-5E  (4IM4)261  AVIIGEFGTIDKN---------NLSSRVAHAEHYAREAVSRGIAVFWWDNGYYNPGDAETYALLNRKTLS
CelCCA  (1EDG)302  PVIIGECGAVDKN---------NLKTRVEYMSYYVAQAKARGILCILWDNNNFS-GTGELFGFFDRRSCQ
CelAcd  (3AYR)281  PMILGEYGAMNRD---------NEEDRATWAEFYMEKVTAMGVPQIWWDNGVFE-GTGERFGLLDRKNLK
                       ▲Glu303                               Trp339▲
                           α8              α6′
AC2a_GH5      361  FIGNGEQAVRAMIDSWENNDPNYTLQSIYDSAPESSR
XG5     (2JEP)349  VT--QQNIINAIMQGMQ--------------------
EngD    (3NDY)328  WD--APEVMKAFIKGIGGSS-----------------
BoGH5A  (3ZMR)447  PH--NAASIAAIMKGLNLEHHHHHH------------
26A-5E  (4IM4)322  WY--YPEIVQALMRGAG--------------------
CelCCA  (1EDG)362  FK--FPEIIDGMVKYAFGLIN----------------
CelAcd  (3AYR)341  IV--YPTIVAALQKGRGLEVNVVH-AIEK--------
```

Fig. S1. Multiple structural alignment by PROMALS3D of *AC2a*Cel5A with its six closest structural homologues. A dark green background indicates identical residues present in >70% of the sequences, whereas a light green colour indicates similar amino acids. Alpha helices and beta-strands present in *AC2a*Cel5A are displayed as red helices or yellow arrows, respectively. Strictly conserved residues in the catalytic centers of GH5 enzymes are indicated by ▲ and the respective residue number in *AC2a*Cel5A. The two catalytic glutamates are also marked by a red asterisk.
