# Supplementary material for: Author Correction: Structural Features of a Bacteroidetes-Affiliated Cellulase Linked with a Polysaccharide Utilization Locus
Source: Sci Rep. 2020 Apr 8;10:6287. doi: 10.1038/s41598-020-62786-2 (PMC7142078; doi:10.1038/s41598-020-62786-2)
Supplement: Supplementary file 1 — Supplementary Information. [file 41598_2020_62786_MOESM1_ESM.pdf]

STRUCTURAL FEATURES OF A BACTEROIDETES-AFFILIATED CELLULASE LINKED WITH A POLYSACCHARIDE UTILIZATION LOCUS

A.E. Naas[1†], A.K. MacKenzie[1], B. Dalhus[2,3], V.G.H. Eijsink[1], P.B. Pope[1†]

1. Department of Chemistry, Biotechnology and Food Science, Norwegian University of Life Sciences, Ås, 1432 NORWAY.
2. Department of Medical Biochemistry, Institute for Clinical Medicine, University Of Oslo, PO Box 4950, Nydalen, N-0424, Oslo, NORWAY
3. Department of Microbiology, Clinic for Diagnostics and Intervention, Oslo University Hospital, Rikshospitalet, PO Box 4950, Nydalen, N-0424, Oslo, NORWAY

†Corresponding Authors: Adrian E. Naas and Phillip B. Pope
Department of Chemistry, Biotechnology and Food Science
Norwegian University of Life Sciences
Post Office Box 5003
1432, Ås
Norway
Phone: +47 6496 6489
Email: adrian.naas@nmbu.no, phil.pope@nmbu.no

Supplementary information

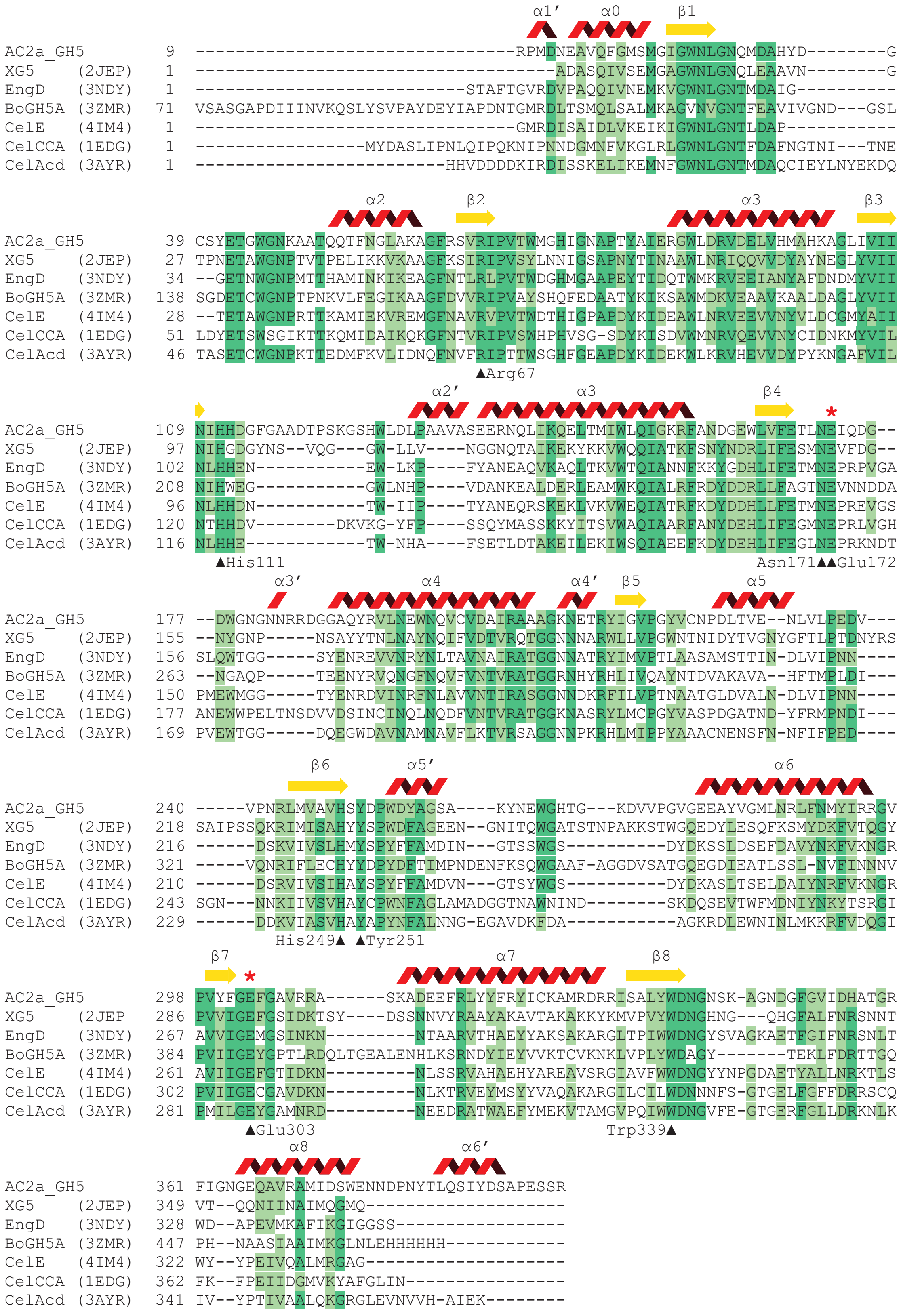

Fig. S1. Multiple structural alignment by PROMALS3D of *AC2a*Cel5A with its six closest structural homologues. A dark green background indicates identical residues present in >70% of the sequences, whereas a light green colour indicates similar amino acids. Alpha helices and beta-strands present in *AC2a*Cel5A are displayed as red helices or yellow arrows, respectively. Strictly conserved residues in the catalytic centers of GH5 enzymes are indicated by ▲ and the respective residue number in *AC2a*Cel5A. The two catalytic glutamates are also marked by a red asterisk.
